# Tamoxifen exacerbates morbidity and mortality in male mice receiving medetomidine anaesthesia

**DOI:** 10.1017/awf.2023.98

**Published:** 2023-12-12

**Authors:** Victoria S Rashbrook, Laura Denti, Christiana Ruhrberg

**Affiliations:** UCL Institute of Ophthalmology, University College London, 11-43 Bath Street, London EC1V 9EL, UK

**Keywords:** adverse effects, anaesthesia, animal welfare, Cre-LoxP, medetomidine, tamoxifen

## Abstract

Tamoxifen-induced CreER-LoxP recombination is often used to induce spatiotemporally controlled gene deletion in genetically modified mice. Prior work has shown that tamoxifen and tamoxifen-induced CreER activation can have off-target effects that should be controlled. However, it has not yet been reported whether tamoxifen administration, independently of CreER expression, interacts adversely with commonly used anaesthetic drugs such as medetomidine or its enantiomer dexmedetomidine in laboratory mice (*Mus musculus*). Here, we report a high incidence of urinary plug formation and morbidity in male mice on a mixed C57Bl6/J6 and 129/SvEv background when tamoxifen treatment was followed by ketamine-medetomidine anaesthesia. Medetomidine is therefore contra-indicated for male mice after tamoxifen treatment. As dexmedetomidine causes morbidity and mortality in male mice at higher rates than medetomidine even without tamoxifen treatment, our findings suggest that dexmedetomidine is not a suitable alternative for anaesthesia of male mice after tamoxifen treatment. We conclude that the choice of anaesthetic drug needs to be carefully evaluated in studies using male mice that have undergone tamoxifen treatment for inducing CreER-LoxP recombination.

## Introduction

The Cre-LoxP system is often used to induce gene deletion in mice, whereby the P1 bacteriophage Cre recombinase (hereafter referred to as Cre) is inserted together with a promoter into the mouse genome to excise genetic material surrounded by engineered LoxP sites in cell types that express Cre (Nagy 2000). CreER is a modified version of Cre, in which Cre is fused to the oestrogen receptor (oestrogen receptor; ER) to retain it in the cytoplasm, but CreER translocates to the nucleus after binding the tamoxifen metabolite 4-hydroxy tamoxifen (4-OHT) (Nagy 2000). Thus, temporally controlled gene deletion is achieved when mice carrying both CreER and floxed target genes are treated with tamoxifen or 4-OHT (Feil *et al.* 1996).

Although CreER-mediated gene modification is now a widely used model to study gene function in the mouse (*Mus musculus*), both tamoxifen and CreER induction have been reported to cause toxicity, with the extent of toxicity dependent on the tamoxifen dose, because tamoxifen itself can be toxic at high concentrations, but also because a higher tamoxifen dose induces more CreER nuclear translocation and thus more off-target effects (Loonstra *et al.* 2001; Bersell *et al.* 2013; Brash *et al.* 2020; Rashbrook *et al.* 2022). For example, the intraperitoneal injection of pregnant dams with tamoxifen at embryonic day (E) 9.75 caused a high incidence of limb abnormalities in E17 mouse embryos (Sun *et al.* 2021). Tamoxifen also causes intrauterine haemorrhage and increases the mortality of pregnant mice injected at E5.5, but tamoxifen treatment of non-pregnant mice does not increase mortality (Ved *et al.* 2019).

CreER-modified transgenic mice may also be used in procedures that require anaesthesia after tamoxifen administration to induce gene deletion, for example, to establish the requirement for specific growth factors in corneal nerve regeneration after injury (Brash *et al.* 2019). The α2-adrenoreceptor antagonist medetomidine or its active enantiomer dexmedetomidine are commonly included in injectable anaesthetic regimes that are used in both veterinary medicine and research studies using live mice (Burnside *et al.* 2013). As dexmedetomidine can cause high and medetomidine low rates of morbidity and mortality in male mice due to urinary tract obstruction by seminal coagulum (Wells *et al.* 2009; Cagle *et al.* 2017), and tamoxifen has been linked to a range of adverse effects on mice including in the testes and spermatozoa (Patel *et al.* 2017), we sought to ascertain here whether animal welfare records accompanying our research studies provided evidence that tamoxifen exacerbated the adverse effects of a medetomidine-based anaesthetic regime in male or female mice.

## Materials and methods

All animal work was carried out with a UK Home Office licence (PP1810839) after ethical and veterinary review through the local Animal Welfare Ethical Review Body (AWERB). Male and female adult mice (more than two months and less than nine months of age) on a mixed C57Bl/6J and 129/SvEv background were housed in individually ventilated GM500 Mouse IVC Green Line cages (391 × 199 × 160 mm; width × depth × height; Tecniplast, London, UK) containing Aspen Chips 2 bedding (Datesand, Stockport, UK), LBS Nest-Pucks, aspen blocks and GLP fun tunnels (LBS Biotech, Horley, UK). Mice of the same sex were group-housed, with a maximum of five mice per cage, and they were fed with a regular chow diet. All mice described in this report were originally intended to provide controls for gene deletion experiments, or used to study the effects of gene deletion with Cre or CreER (Brash *et al.* 2019). Mice given medetomidine anaesthesia and enrolled in experiments scheduled to persist for at least seven days post-anaesthesia were included; in total, 100 animals were studied. Day 0 was defined as the day of anaesthesia. As per local recommended anaesthetic practice to induce deep anaesthesia prior to surgery, mice were then given 75 mg kg^–1^ of ketamine (Narketan; Vetoquinol, Towcaster, UK) and 0.5 mg kg^–1^ of medetomidine (Dormitor; Vetoquinol) via intraperitoneal injection, both diluted in sterile water. Intraperitoneal injection of 1 mg kg^–1^ atipamezole diluted in sterile water (Antisedan; Zoetis, Leatherhead, UK) was used to resolve medetomidine action. Mice were kept on a warming pad until fully recovered from sedation. Mice were returned to their home cages and monitored daily for seven days after anaesthesia. Eighteen male and six female mice were not injected with tamoxifen whereas other mice in the same study were injected intraperitoneally on two occasions (dose 1 and dose 2) with 0.5 mg tamoxifen (Merck, Gillingham, UK) dissolved in 85 μl or 250 μl vegetable oil (Merck); this tamoxifen dose was chosen as a reported effective dose to induce genetic deletion in adult mice with Cagg-CreER^TM^ (Brash *et al.* 2019). In some studies, mice were injected 7 and 3 days or 7 and 0 days before anaesthesia; when tamoxifen was given on the same day as the anaesthesia was induced (day 0), tamoxifen was given first (together, 12 males and 17 females). In other studies, mice were injected 28 and 14 days prior to anaesthesia (32 males and 15 females). Mice were monitored daily for clinical signs such as hunching and grimacing, mobility and signs of a hard or distended bladder. Mice were euthanased by an appropriate schedule 1 method at the end of the experiment or upon reaching their humane endpoints. Humane endpoints were defined as an inability to urinate, severely reduced mobility or signs of distress or pain as evidenced by the grimace scale. All data analysed here were obtained from mice used for pilot studies designed to test the effect of gene deletion on cardiovascular outcomes, including appropriate controls. Accordingly, the mice analysed here were pooled from several such pilot studies and did not themselves comprise a designed toxicity study; specifically, mice had not been randomised into uninjected and tamoxifen-treatment groups. All authors were aware which groups of mice had received tamoxifen treatment and which had not, both during weight measurements and recording of the onset of adverse effects, because the mice had originally been allocated to pilot studies testing the effect of gene deletion, rather than testing whether there is an interaction of tamoxifen treatment with subsequent anaesthesia.

### Statistical analysis

This was performed using GraphPad Prism v9.2. Survival curves were analysed for differences using a Log-rank (Mantel-Cox) test. *P*-values of ≤ 0.05% were considered significant.

## Results

**Figure 1.**
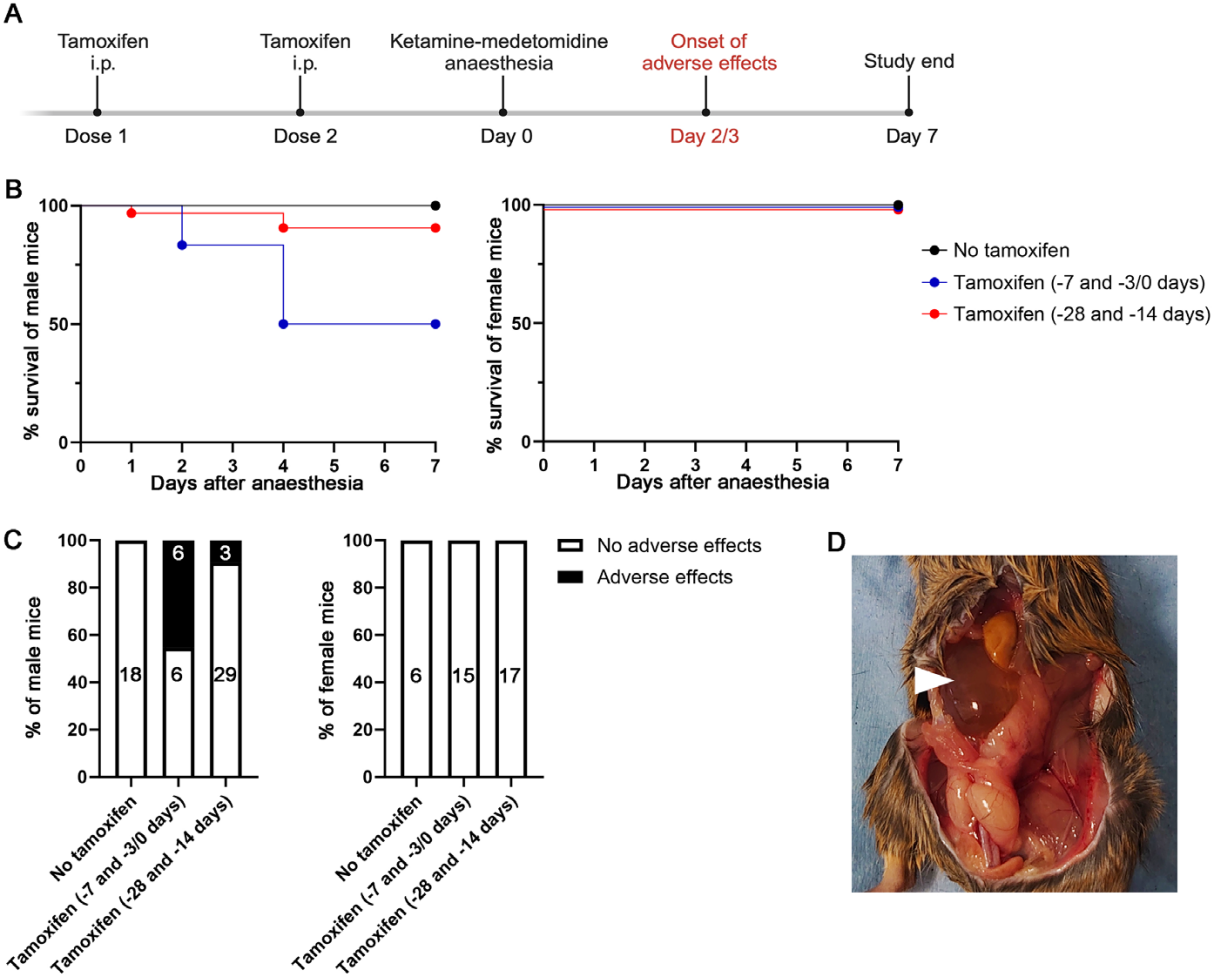
Showing (A) Treatment and analysis timeline: Day 0 is defined as the day of anaesthesia with ketamine-medetomidine. In some studies, mice were injected with tamoxifen on day 7 (dose 1) and either on day 3 or 0 (dose 2) prior to anaesthesia (day –7 and –3/0). When the second tamoxifen dose was given on day 0, tamoxifen was given prior to anaesthesia. In other studies, mice were injected intraperitoneally with tamoxifen on days 28 (dose 1) and 14 (dose 2) prior to anaesthesia (day –28 and –14). All studies were terminated seven days after anaesthesia. (B) Survival curves of male and female mice up to seven days after anaesthesia. Male mice injected with tamoxifen on day –7 and –3/0 prior to anaesthesia had significantly reduced survival (six out 12 male mice lost) compared to uninjected (no tamoxifen) male mice (0 out of 18 male mice lost); *P* = 0.0009. Male mice injected with tamoxifen on day –28 and –14 prior to anaesthesia had a lower rate of adverse effects (three out of 32 male mice lost); this observation was not statistically significant at the number of mice included in our studies (*P* = 0.1861). No female mice were lost. (C) Percentage of male and female mice with adverse versus no adverse effects within seven days of anaesthesia, after mice had received no tamoxifen or tamoxifen on day –7 and –3/0 or day –28 and –14 schedule. Black numbers on the white bars indicate the number of mice without adverse effects and white numbers on the black bars indicate the number of mice with adverse effects. (D) Example of a male mouse culled due to severe adverse effects, sprayed with ethanol before autopsy to prevent fur-shedding; the arrowhead indicates a distended bladder.

Adult male and female mice were either uninjected or injected intraperitoneally with 0.5 mg tamoxifen on two occasions prior to anaesthesia with 75 mg kg^–1^ ketamine and 0.5 mg kg^–1^ medetomidine and, to resolve anaesthesia, 1 mg kg^–1^ atipamezole (Figure 1A). Mice injected with tamoxifen were dosed with ketamine-medetomidine anaesthesia either 28 and 14 days, or 7 and either 0 or 3 days prior to injection. Mice were monitored twice daily for adverse effects. We unexpectedly observed that male mice injected with tamoxifen had an increased incidence of severe adverse effects 48–72 h after ketamine-medetomidine anaesthesia. Moreover, male mice with the second tamoxifen dose three days prior or on the day of anaesthesia induction had a 50% incidence of adverse effects compared to 9.4% in mice that had received their second tamoxifen dose 14 days prior to anaesthesia, and male mice not given tamoxifen had no adverse effects (Figure 1B, C). Severe adverse effects were seen in both male mice expressing CreER and in CreER-negative control male mice, suggesting that the adverse effects were exhibited regardless of whether the mice also had a genetic deletion. Moreover, female mice showed no obvious adverse effects, regardless of their tamoxifen dosing schedule (Figure 1B, C). As prior reports had suggested that medetomidine can cause morbidity and mortality in 3% of male mice due to urinary tract obstruction by seminal coagulum (Wells *et al.* 2009; Cagle *et al.* 2017), male mice found at their humane endpoints were scruffed for abdominal examination and, in some cases, were found to present with a hardened region in the abdomen and were always unable to urinate, which is a normal response to scruffing in healthy mice. Of the male mice euthanased at their humane endpoint, three were analysed post mortem for gross abnormalities, and all were found to have distended bladders when the abdominal cavity was opened (Figure 1D). Some mice were found dead, thereby exceeding the pre-specified humane endpoint of the study, requiring the filing of a Home Office UK standard condition 18 report, which was sent to the named animal welfare office, local veterinarian and legislator. Specifically, we observed a significantly decreased survival rate up to 72 h post-anaesthesia in male mice injected with tamoxifen compared to male mice not injected with tamoxifen (*P* > 0.001) (Figure 1B). By contrast, female mice did not have decreased survival after tamoxifen dosing followed by anaesthesia (Figure 1B).

## Discussion

Our study has established that the combination of tamoxifen injection with the anaesthetic agent medetomidine increased mortality in male mice 24–72 h post-anaesthesia. Medetomidine is comprised of a combination of two enantiomers, dexmedetomidine and levomedetomidine, whereby dexmedetomidine is thought to be the active form and typically used at half the dose of medetomidine (Burnside *et al.* 2013). Both medetomidine and dexmedetomidine have been suggested as comparable in anaesthetic effect, and each needs to be combined with ketamine for deep anaesthesia (Burnside *et al.* 2013). Even though medetomidine and dexmedetomidine are commonly used in both veterinary medicine and research with live mice (Burnside *et al.* 2013), a literature search after observing adverse effects identified two published studies reporting that medetomidine and dexmedetomidine cause mortality in C57Bl/6J male mice at an incidence of 3 and 67%, respectively (Wells *et al.* 2009; Cagle *et al.* 2017). In agreement with the lower incidence of 3% mortality with medetomidine compared to dexmedetomidine, we did not observe any deaths in the small cohort of male mice that had received ketamine-medetomidine but no other intervention. By contrast, we observed increased mortality in 50% of male mice treated with tamoxifen within three days and in 9.4% of male mice treated with tamoxifen within 14 days of receiving medetomidine. These findings suggest that tamoxifen exacerbates medetomidine toxicity and that a shorter interval between administering both drugs is the most detrimental.

Medetomidine has been used in a range of domestic animal species for veterinary semen collection with a catheter under anaesthesia and is associated with increased sperm counts (Zambelli *et al.* 2008; Madrigal-Valverde *et al.* 2021). In accordance with this, prior work reporting increased mortality in male mice linked this to the formation of seminal coagulum that blocked the urethra (Wells *et al.* 2009), presumably because ejaculation was stimulated but the ejaculate was not released or manually removed, as is the case in veterinary practice for semen collection. As previously described for medetomidine-anaesthetised male mice in a study not using tamoxifen (Wells *et al.* 2009), the medetomidine-anaesthetised male mice treated with tamoxifen in our study also had distended bladders on post mortem. Nevertheless, it is unclear why tamoxifen treatment increased the incidence of this medetomidine-induced adverse effect. As tamoxifen treatment in male mice decreases the proportion of mature elongated sperm cells at the expense of rounded, immature spermatogonia in the Sertoli cells (Patel *et al.* 2017), it is conceivable that immature sperm cells released into the seminal fluid may increase the incidence of urethral plug formation.

As this study is an observational report, there are certain limitations. Firstly, a number of mice included in this report would have undergone gene recombination at the intended Cre targets to induce gene deletion, which may have exacerbated or increased the incidence of observed adverse effects; however, adverse effects were also seen in tamoxifen-injected males lacking Cre target sites and thus gene deletion, arguing against gene targeting contributing to the adverse effects attributed to the anaesthetic regimen. Secondly, vehicle-treated mice were not included in the groups of mice receiving medetomidine anaesthesia and, therefore, we cannot formally exclude that the vehicle rather than tamoxifen caused the observed adverse effects. However, the observation that males were selectively affected argues for an adverse hormonal effect due to tamoxifen. Thirdly, it cannot be excluded that elements of the anaesthetic regimen other than medetomidine increased mortality in tamoxifen-treated mice, or that mechanisms other than seminal coagulum are responsible; however, both possibilities seem unlikely, because female mice did not show mortality in our study. Moreover, a prior report showed that medetomidine toxicity in male mice was mitigated by replacing medetomidine with xylazine, whilst retaining ketamine and atipamezole in the anaesthetic protocol (Wells *et al.* 2009). Further re-analysis of existing or ongoing studies carried out in other labs with other anaesthetic regimens would be required to determine whether tamoxifen can also exacerbate the side-effects of other anaesthetic regimens. We trust that the report would precipitate such analysis to improve animal welfare in Cre-LoxP experiments.

Notably, the mortality and morbidity associated with tamoxifen use prior to medetomidine-based anaesthesia decreased with a longer duration between the last tamoxifen dose and the induction of anaesthesia. Thus, mortality in male mice was reduced from 50 to 9.4% when the last dose of tamoxifen was administered 14 days before anaesthesia, as opposed to 0 or 3 days before anaesthesia. Nevertheless, we consider a 10% mortality incidence unacceptable. An even longer duration between tamoxifen administration and induction of anaesthesia may further reduce the mortality rate but may be unsuitable for some experimental models due to recovery from the intended purpose of tamoxifen administration, which is to induce gene deletion. Thus, a population of recombination-resistant cells may have an advantage over recombined cells and out-compete them (e.g. Fantin *et al.* 2013). Therefore, we suggest that alternative injectable anaesthetic formulations not containing medetomidine should be used when using male mice, particularly in combination with tamoxifen administration to induce gene deletion, and when inhalation anaesthesia is unsuitable.

### Animal welfare implications

This report seeks to document and alert other researchers to the adverse effect of tamoxifen administration prior to medetomidine anaesthesia in male mice. The significant morbidity and mortality we have observed constitute ethical reasons that precluded us from including additional animals in a thoroughly designed experimental study with larger group sizes. Nevertheless, our report should significantly impact animal welfare by prompting researchers to avoid medetomidine and dexmedetomidine for anaesthesia of tamoxifen-treated mice in genetic recombination studies using Cre-LoxP. This is particularly important, because both tamoxifen and medetomidine are commonly injected into mice for *in vivo* experiments to study gene function in injury and disease models seeking to provide novel information relevant to human health, but alternative anaesthesia protocols are available for which adverse effects have not been reported.
